# Monitoring and Identification of Road Construction Safety Factors via UAV

**DOI:** 10.3390/s22228797

**Published:** 2022-11-14

**Authors:** Chendong Zhu, Junqing Zhu, Tianxiang Bu, Xiaofei Gao

**Affiliations:** School of Transportation, Southeast University, Nanjing 211189, China

**Keywords:** road construction, safety, unmanned, aerial, vehicle, convolutional neural network

## Abstract

The safety of road construction is one of the most important concerns of construction managers for the following reasons: long-span construction operation, no fixed monitoring cameras, and huge impacts on existing traffic, while the managers still rely on manual inspection and a lack of image records. With the fast development of Unmanned Aerial Vehicle (UAV) and Artificial Intelligence (AI), monitoring safety concerns of road construction sites becomes easily accessible. This research aims to integrate UAVs and AI to establish a UAV-based road construction safety monitoring platform. In this study, road construction safety factors including constructors, construction vehicles, safety signs, and guardrails are defined and monitored to make up for the lack of image data at the road construction site. The main findings of this study include three aspects. First, the flight and photography schemes are proposed based on the UAV platform for information collection for road construction. Second, deep learning algorithms including YOLOv4 and DeepSORT are utilized to automatically detect and track safety factors. Third, a road construction dataset is established with 3594 images. The results show that the UAV-based monitoring platform can help managers with security inspection and recording images.

## 1. Introduction

Road infrastructure is a fundamental component of the modern transportation system and has a substantial influence on the economy. The construction of a roadway is one of the most important processes as it determines the quality as well as the long-time performance of the roadway. There are many factors to consider during the roadway construction project, among which the safety factor is of special concern as it involves human activity with heavy machines. It is necessary to monitor the safety risks of roadway construction and provide warnings in advance.

Currently, many potential safety hazards exist in road construction which will be detailed below. First, road construction generally has an impact on the existing traffic, and optimizing the traffic flow is important for safety management. However, conventional vertical road signs and a flagman constitute huge risks in the construction process [1,2]. Dobromirov et al. [3] proposed a safety assessment including construction dump trucks. Generally, construction work and normal traffic operate together, and they will affect each other. Therefore, the situation at the road construction site is truly complex. Second, the road construction operation area has a long span and long construction time cycle. The traditional manual inspection cannot quickly carry out a comprehensive safety investigation on the construction operation area, and some safety factors may be ignored. Finally, due to the moving characteristics of road construction, it is troublesome to install fixed cameras for building construction, so there is a lack of safety monitoring in the construction process. Nkurunziza [4] conducted a safety survey on road construction in Rwanda, and the results showed that the level of accidents caused by lack of safety recorded at the road construction site is still high, 78.57%. In addition, the safety climate will affect the safety management of the road construction site because road construction operations are scattered, which makes it difficult to implement the safety policy correctly [5,6]. Accordingly, many authors have studied how to use new technologies to reduce exposure to accidents and improve construction security. Zadobrischi and Dimian [7] designed a road safety intelligent system based on future cars and pedestrians, analyzed the approach leading to accidents and the classification of situations, and verified it in several traffic scenarios. A safety monitoring platform [8,9,10,11,12,13] is an effective method. It can quickly collect and analyze complex information on the construction site and alert “abnormal” behavior, reducing the possibility of accidents. This research proposes a safety monitoring platform for road construction based on Unmanned Aerial Vehicles (UAVs).

Unmanned Aerial Vehicles (UAVs) are developing rapidly, and various technologies, such as remote control, autonomous cruise, battery life, and equipped sensors have gradually matured, which has greatly improved the controllability, stability, flexibility, and endurance of UAVs [14,15,16]. Yu et al. [17] proposed a UAV path-planning method to realize the unknown exploration efficiently; a Rapidly-exploring Random Tree (RRT) incrementally built a topological road map that provides the waypoints of an optimal informative path. Consequently, UAV technologies are widely used in various industries, including construction, agriculture, electricity, and so on [18,19,20,21,22,23]. This research mainly studies the application of UAVs to the monitoring and identification of safety factors for road construction.

In addition, Artificial Intelligence (AI) technology can be applied to realize the automatic monitoring and identification of safety factors. Deep learning algorithms, especially convolutional neural networks, are increasingly popular in the field of computer vision, which can quickly detect and track targets, mainly pedestrians and vehicles [24]. Target detection [25,26] is one of the most basic and important problems in computer vision. Therefore, many authors study target detection algorithms based on deep learning [27,28,29]. Convolutional neural networks [30,31] are one of the most widely used and effective tools. The structure of a convolutional neural network mainly includes an input layer, a CONV layer, a ReLU layer, a Pooling layer, an FC layer, and other structures. YOLO (you only look once) is a new target detection algorithm, which can realize real-time, fast, and accurate target detection. The process of target detection is unified into a single neural network. The neural network uses the whole image information to predict the bounding boxes of the target, identify the category of the target at the same time, and realize the end-to-end real-time target detection task. YOLOV4 [32,33,34,35,36] is one of the updated algorithms to achieve faster detection speed and higher accuracy. Multi-target tracking [37,38,39,40] is also one of the problems in computer vision research. Its algorithm mainly consists of detector and tracker. However, there is no detection algorithm specially applied to UAV aerial video. Given that the shooting angle of UAVs is different from that of traditional cameras, the detection accuracy of aerial video is not high. Many authors have done a lot of research on this. Chen X. [41] et al. and Ke R. [42] et al. put forward their algorithms, mainly to improve the detection accuracy in complex traffic environments and deal with the influence of UAV irregular motion. Wang et al. [43] introduced the concept of quantity into the deep learning model, and Baidu and Jeong [44] added ConvMixers to the prediction head based on YOLOv5 architecture for solving the challenge of minutely small objects.

**Figure 1:**
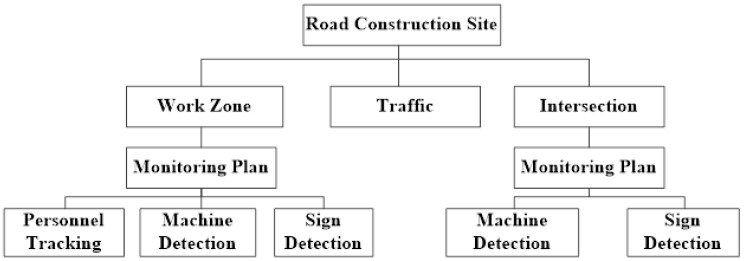
The Framework of UAV-based road construction security monitoring platform.

In general, many papers have researched road construction safety in two aspects: organization management and safety climate [1,2,3,4,5,6]. It is rare to integrate Artificial Intelligence (AI) technology and UAV technology into road construction safety, which would provide managers with more rapid inspection and more comprehensive safety information. Considering the security problems existing in road construction mentioned above, this research proposes a road construction safety monitoring scheme based on UAVs. The framework of this study is presented in Figure 1. The objective of this study is to monitor road construction and the of identification the safety factors which may cause construction accidents. This paper proposes a monitoring framework based on the UAV, including the monitoring plan and the UAV flight schemes in different zones. It also realizes automatic detection and tracking of multiple objects, greatly improving the efficiency of security inspection. At the same time, a data set of road construction scenes has been established, including 3594 pictures. Lastly, the UAV monitoring platform is applied to road construction in Nantong City, and the results show that the method can effectively improve safety inspection efficiency and management.

## 2. Monitoring Framework

The main purpose of this study is to establish a road construction safety monitoring platform based on UAVs. This chapter mainly introduces the specific monitoring framework. On the one hand, corresponding monitoring plans are designed for different areas of the road construction site, including UAV flight mode and parameter design; on the other hand, the content of monitoring is changed in different construction processes and the safety decisions are made based on the results.

### 2.1. Partition of Construction Site

**Figure 2:**
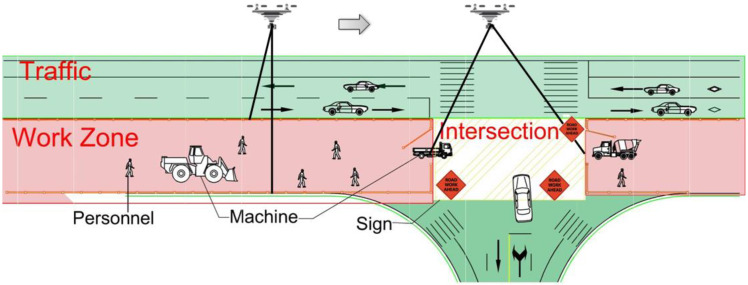
Partition of the road construction site.

The road construction site is a complex scenario composed of many different objects and areas. To monitor the road construction site, it is necessary to partition the entire area into specific areas based on their functions. The UAV is utilized first to scan the entire work zone and obtain images of the entire area. The road construction site is then partitioned into the work zone, traffic area, and cross area. While the road infrastructure typically expands over a long distance, the construction site is typically restricted to a small area, as illustrated in Figure 2. The red area is the work zone, where the paving machines and personnel locate, and is the main focus. The green area is the traffic area, where the road is open to normal traffic. Since the lane under construction is parallel to the lane of traffic, a guardrail needs to be set between them to separate construction from traffic. The intersection is where the work zone and traffic intersect, and work zone signs are needed in this area.

#### 2.1.1. Work Zone Monitoring Plan

The work zone is of primary focus for safety monitoring as workers and paving machines are located in this area. The objective of this part is to monitor the activities of personnel and machines and to identify safety concerns through their trajectories. The flight route of the UAV is illustrated in Figure 2 and specific flight parameters are discussed in the next section.

#### 2.1.2. Intersection Monitoring Plan

The intersection area is the other complex area as regular traffic and construction vehicles intersect in this area. The objective of this part is to monitor vehicles that enter and leave this area. Due to the flight mission, the UAV just needs to hover over the Cross Area for covering the whole intersection when construction vehicles are driving in the Cross Area. The flight altitude is 15 m. UAV basic parameters are shown in Table 1 and specific flight parameters are described in Table 2. When the vehicles drive in or out of the Cross Area, the risk of these vehicles being exposed to accidents is significantly increased. Normally, there are no traffic lights in the Cross Area and the existing traffic will be interrupted when construction vehicles merge into the normal traffic flow. In particular, most construction vehicles are large vehicles such as trucks. Consequently, these vehicles will be defined as ‘abnormal’ and can be identified automatically by multi-target track algorithms. Furthermore, this makes up for the lack of intersection monitoring due to road construction.

### 2.2. UAV Settings

To obtain the best UAV videos containing comprehensive construction information, the UAV flight settings need to be designed in different construction areas because of the various flight missions. The UAV flight parameters include flight altitude, flight speed, and camera parameters whose tuning process is presented below. The UAV used in this research is DJI Mavic 2. The UAV’s basic parameters are shown in Table 1. The fundamental objective of road construction security management is to distinguish complex factors on road construction sites that contain constructors, construction vehicles, normally running cars, etc.

#### 2.2.1. Flight Altitude

Due to the fixed focal length of the camera, the flight altitude affects the shooting range, while construction areas should be photographed comprehensively. Different flight altitudes are required for different construction areas for unique flight missions. The relationship formula between the shooting range and flight altitude is described by Equation (1).

(1) $$\frac{f}{H}=\frac{a}{D}\;$$

where *H* is the flight altitude, f is the focal length of the camera, a is the camera sensor size, and *D* is the shooting range. Based on the parameters of the UAV, the focal length is 28 mm and a is 12.8 mm while the camera sensor size is 12.8 mm × 9.6 mm. This research takes the construction of a four-lane road as a field validation. The construction is carried out with half the road width. If the flight mission is acquiring an aerial view of the whole road, *D* should be chosen as 17 m and the flight altitude is calculated as 13 m while 15 m can be chosen as the final flight altitude. If the flight mission just focuses on the construction area, *D* should be chosen as 7.5 m and the flight altitude calculated as 5.9 m while 10 m can be chosen as the final flight altitude.

#### 2.2.2. Flight Speed

The data collected by UAVs in this research are mainly video, so there is no influence between flight speed and shutter speed. However, the influence of UAV endurance and flight speed needs to be considered due to the long distance of road construction. If the flight mission is to obtain the whole length of construction, the UAV needs to select the flight speed with the maximum range while the maximum range is 18 km with the 50 km/h constant flight speed based on the UAV basic parameters. If the flight mission is to photograph the construction process, the flight speed should not be too fast for obtaining the position and trajectory of all construction personnel and vehicles.

### 2.3. UAV Monitoring and Safety Management Decision-Making

**Figure 3:**
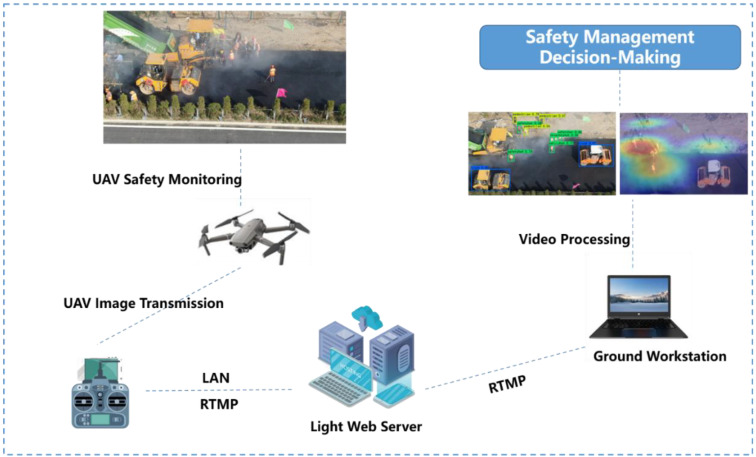
Implementation of UAV safety monitoring.

**Figure 4:**
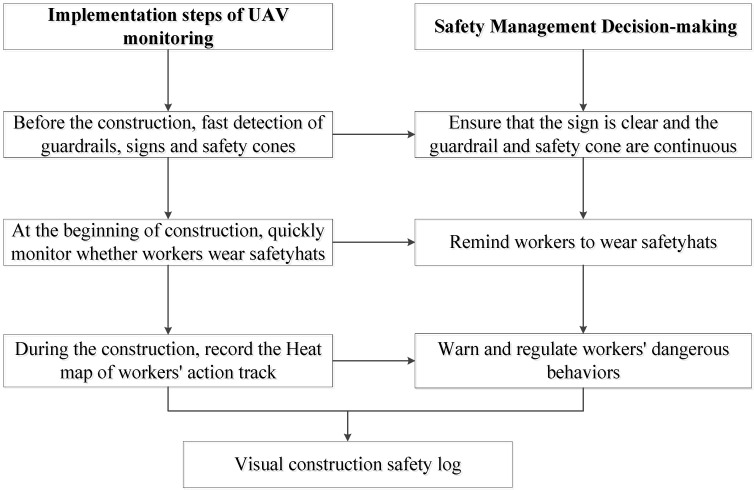
Steps of safety management decision-making based on the UAV.

This research realizes the rapid detection of the comprehensive information that is difficult to obtain manually during the construction process through UAV and provides more accurate and detailed information for safety management decision-making that better prevents the occurrence of accidents. The data collected by the UAV is transmitted to the mobile terminal and PC terminal by wireless image transmission, and the PC terminal processes the data at the same time. Figure 3 and Figure 4 show the implementation of UAV monitoring and the steps of the safety management decision-making based on the UAV.

## 3. Deep Learning Algorithms

Object-detection and target-tracking deep learning algorithms were used in this study to identify and track personnel, machines, and vehicles. The following section briefly describes the algorithms.

### 3.1. Object Detection Based on YOLOv4

Objects in the Work Zone mainly include constructors, vehicles, and signs. In addition, some constructors may not wear safety hats which should also be identified. In this study, YOLOv4 was selected as the target detection algorithm for the identification of objects in the Work Zone.

YOLOv4 is a target detection algorithm that pays more attention to the detection speed with high accuracy. Bochkovskiy A et al. [36] points out that the significance of the work is enhancing the operating speed of an object detector in production systems and optimization for parallel computations. The majority of exact conventional neural networks can not realize real-time operation, and many GPUs are required for training with a large mini-batch-size. Instead, YOLOv4 figures out these problems, which operating in real-time, and training just needs only one GPU.

**Figure 5:**
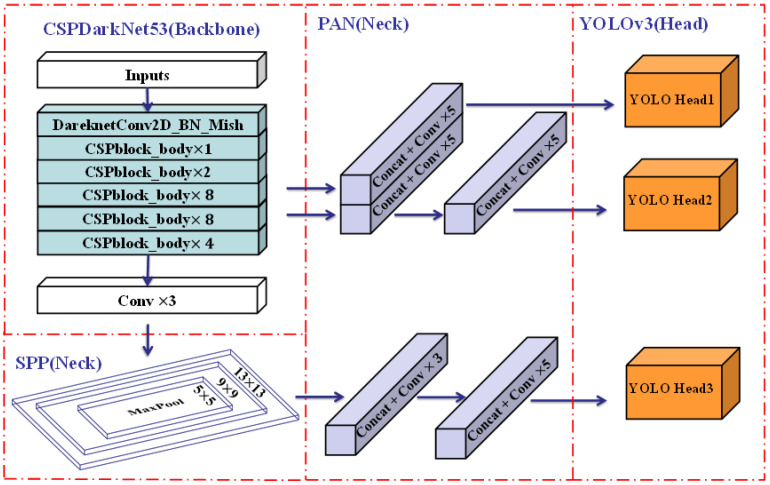
Architecture of YOLOv4. CSPDarknet53 (Backbone): Cross Stage Paritial Network. SPP(Neck): Spatial Pyramid Pooling. PAN(Neck): Path Aggregation Network.

YOLOv4 has an architecture of a detector that consists of a CSPDarknet53 backbone, an SPP additional module, a PANet path-aggregation neck, and a YOLOv3 [45] (anchor-based) head. The reason for choosing this architecture is the detector’s higher input network size, and more layers. In terms of additional blocks, it aims to enhance the receptive field and integrate different parameters, while the SPP additional module and PANet path-aggregation neck satisfy these objectives. On the other hand, the options of ‘bag of freebies’ and ‘bag of specials’ are also significant for object detection training. YOLOv4 eliminates the training activation function because whether it is PReLU and SELU or ReLU6, they all fail to meet simple training. The regularization function is DropBlock, which is the best method when compared with others. In terms of normalization, syncBN is removed since only one GPU is used for training. Finally, there are still other approaches for increasing the function of the neural network. YOLOv4 upgrades the data augmentation called Mosaic; four images are mixed for training which has a large significance for the dataset. Besides that, Self-Adversarial Training (SAT) is also a new option for data augmentation. Excellent hyper-parameters have been chosen to apply genetic algorithms. In addition, YOLOv4 improves many modules for training and detecting more adequately, including SAM, PAN, and Cross mini-Batch Normalization (CmBN). Generally, the architecture of YOLOv4 is shown below in Figure 5.

### 3.2. Tracking Heat Map Generation Based on YOLOv4-DeepSORT

To ensure the safety of constructors and prevent accidents, the activities of constructors in the Construction Area should be monitored. Therefore, this research tracks constructors by the UAV monitoring videos that are, also, automatic. Tracking heat maps are generated based on the constructor activities, where red indicates high activity and blue indicates low activity.

Multiple object tracking is also one important aspect of computer vision, which belongs to Artificial Intelligence (AI). The algorithm of multiple object tracking is composed of two parts: the detector is YOLOv4, and the tracker applies the DeepSORT [46] tracking technique.

**Figure 6:**
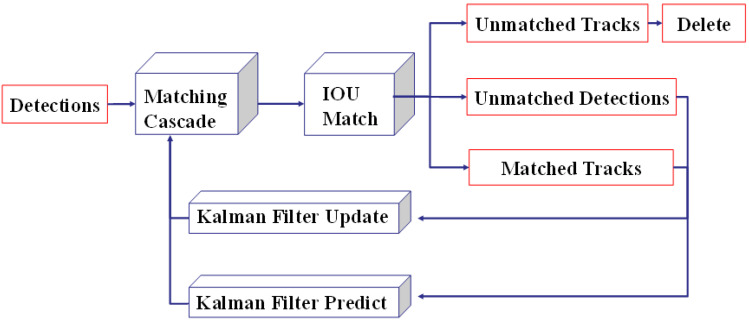
Architecture of DeepSORT.

Simple Online and Realtime Tracking (SORT) [47] is a popularly adopted multiple object-tracking algorithm that has effective performance on simple object tracking. It contains a Kalman filter and a Hungarian algorithm, where the object position is predicted by the Kalman filter and the bounding box will be measured with an association metric using the Hungarian. Wojke N et al. [46] extended SORT with a deep association metric called DeepSORT to improve its performance. DeepSORT has a convolutional neural network (CNN) to pre-train object detection on the re-identification dataset. In other words, the accuracy of object detection is enhanced by CNN, which can be trained offline. Therefore, DeepSORT performed better in scenes where occlusions appear by using less computational power when the image information has been integrated into motion. The general architecture of DeepSORT is shown in Figure 6. In particular, DeepSOCIAL [40] is one of the valuable practices used to track pedestrians for automatic monitoring of social distance when COVID-19 is raging all over the world and endangering human health.

## 4. Training and Evaluation

This chapter mainly introduces the establishment of the road construction data set, the adjustment of parameters for training, and the evaluation of training results.

### 4.1. Dataset

This research produces a dataset of road construction, and the objects include constructors, construction vehicles, construction signs, and guardrails. Furthermore, whether constructors wear safety hats will also be detected. The images of the dataset mainly include the cutting of UAV videos and downloading from the Internet. Then, the dataset is augmented through the flip, rotation, scale changing, cropping, translation, and Gaussian Noise of pictures. Finally, the total image of the dataset is 3594. In addition, YOLOv4 [36] adopts a new augmentation method called Mosaic that mixes 4 training images for better accuracy. Labeling is selected as the annotation tool of the picture, which is popular in the production of datasets.

### 4.2. Training Parameters

Although the deep learning algorithm has been relatively perfect, there are still some problems when training the model because a target detection model for road construction has not been developed. To improve the accuracy and efficiency of training, the parameters need constant tuning. The specific parameters will be described below.

#### 4.2.1. Pre-Trained Weights

First, transfer learning is used to advance the performance of the model. Transfer learning means accelerating the feature extraction that benefits from the pre-trained weight. The new model can learn new things based on existing models just like people learn new knowledge based on what they have learned before. In other words, the road construction model can achieve better learning performance based on well-trained models even if the dataset is small. This research chooses YOLOv4-weights as the pre-trained weights.

#### 4.2.2. Anchor Size

The anchor box means the boxes set in advance on images for learning the features of the target by sliding these boxes. The target to be detected may appear in any position of the image, and the target may be of any size and shape. The position of the target is determined by the points of the feature map extracted by the Convolution Neural Network (CNN). The size of the target is represented by the scale of the anchor box, and the shape of the target is represented by the aspect ratio of the anchor box. Traditionally, the scale and aspect ratio of the anchor box are preset such as Faster-RCNN and SSD. However, the size of the anchor box and error are interrelated. Given that the size of the anchor box is different, the error of the large box may be larger, and the error of the small box may be smaller. This imbalance makes it difficult to judge the clustering. Different from other deep learning algorithms, YOLOv4 does not use the combination of a preset aspect ratio and scale but uses the K-means clustering method to learn anchor boxes from the annotations of the dataset. Therefore, the size of the anchor boxes is independent of the error. The size of anchor boxes in the road construction model is calculated and shown in Figure 6.

#### 4.2.3. Parameters

To improve the performance of this model, the training parameters need to be adjusted. Finally, these parameters are described in Table 3.

### 4.3. Evaluation Metrics

**Figure 7:**
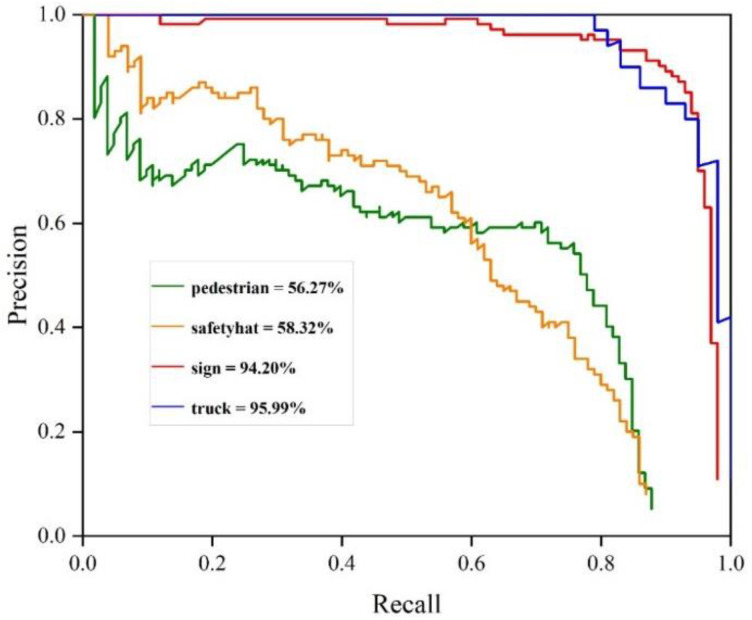
AP of the model.

Mean Average Precision(mAP) and Intersection over Union (IoU) are adopted as the evaluation metrics. Intersection over Union (IoU) means the ratio of the intersection and union of the predicted and ground truth. Precision represents the probability that the predicted positive sample is actually a positive sample. Recall represents the probability of predicting positive samples in actual positive samples. It is generally believed that the prediction is correct only when the IoU of the predicted frame and ground truth are greater than 0.5 (classified as TP). Mean Average Precision(mAP) is the mean value of average precision. The average precision (AP) of each class is shown in Figure 7 and the mAP is 76.19%.

(2) $$\mathrm{Percision}=\frac{\mathrm{TP}}{\mathrm{TP}+\mathrm{FP}}$$

## 5. Field Validation

This chapter is mainly about the practical application of the UAV monitoring platform proposed in this research. The field experiment results show that the monitoring method can effectively identify targets and track workers, which is a supplement to the image data of road construction.

### 5.1. General Information

The road construction field was chosen as a two-way four-lane road in Nantong which is under maintenance. This research collected videos by DJ Mavic 2 including the progress of construction and the whole field of the road. The videos of the experiment were collected for about 30 min.

### 5.2. Object Detection Results

**Figure 8:**
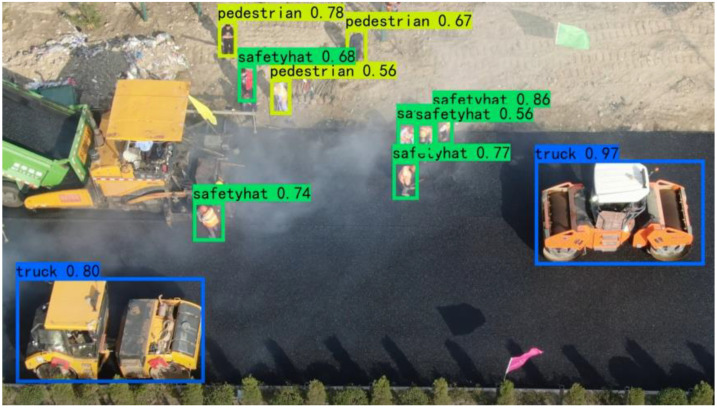
Monitoring of the road construction site.

To ensure the safety of road construction, many objects are the concern of managers that can be automatically identified by YOLOv4. These objects include constructors, construction vehicles, signs, and guardrails. In addition, whether workers wear safety hats will also be detected which is essential for the security of constructors. Security managers can quickly obtain the concerned information about these objects on the mobile terminals and take timely measures to avoid accidents. The results of object detection are shown in Figure 8.

### 5.3. Tracking Heat Map Results

**Figure 9:**
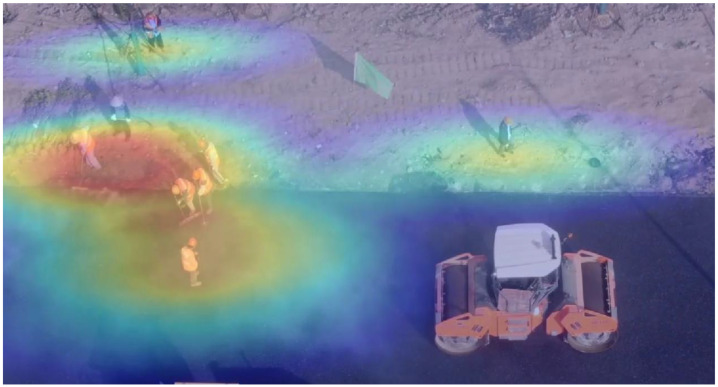
Tracking heat map of constructors.

The safety of constructors is the top priority of construction management and should be paid more attention. Therefore, this research can track the trajectory of constructors and give an early warning of dangerous behaviors. The expression trajectory tracking adopts is the tracking heat map, which can more clearly show the trajectory of constructors. The redder the tracking heat map, the more active the constructors are. The blue area in the track heat map indicates that the workers have not been there. If the red area overlaps with the construction vehicles, it indicates that there are potential safety hazards in the construction process. The results are shown in Figure 9. The construction manager can obtain the frequent activity range of workers from the heat maps, to regulate some dangerous behaviors of workers, such as exceeding the construction area and staying in the direction of construction vehicles, reducing the probability of accidents caused by human factors, and improving the safety of the construction site.

### 5.4. Discussion

Obviously, the UAV-based road construction monitoring platform solves many security management problems in road construction. First, the Construction Area and Traffic Flow Area must be completely separated for normal traffic not to be affected by construction. In this research, the method of UAV aerial photography is proposed to quickly divide the Construction Area and Traffic Flow Area, and automatically detect the signs and guardrails to ensure that they are not missing or damaged. Second, the regional span of road construction is very long, which leads to safety managers spending a lot of manpower and material resources to obtain information on the construction field. However, this problem can be easily solved by using UAVs which can collect construction information through aerial photography. Finally, the monitoring and identification of safety factors based on UAV photography is the detailed and accurate information basis that managers can use to make security decisions and a supplement the lack of safety monitoring in the road construction field.

## 6. Conclusions

UAV-based road construction monitoring platforms plays a large role in the safety management of road construction. The scenarios used by UAVs in this article comply with local UAV control policies. The contributions of this research are as follows: (1) This research establishes a safety management platform for road construction based on UAV photography and a set of UAV flight schemes is proposed, which is good for the intellectualization of construction. (2) Automatic detection and tracking methods are realized through a combination of YOLOv4 and DeepSORT, which greatly improve the efficiency of obtaining construction safety information. (3) The road construction field images can be successfully collected by the UAV platform. These images have been processed and a dataset of 3594 images in road construction is established. The dataset [48] is available as open-source data for the community.

The advantages of the UAV-based monitoring platform mainly include the following points. First, the flexible characteristics of UAVs greatly improves the efficiency of safety inspection at the construction site and reduces the risks faced by inspectors. Second, it makes up for the lack of image records on the road construction site. The target detection and tracking algorithm improves the efficiency of the security manager in obtaining the on-site target information and reduces manual pressure.

Although the proposed platform demonstrates good performance in field tests, there are some limitations to this research. On the one hand, the UAV-based monitoring platform is affected by the attributes of the UAV itself including stability and endurance. Moreover, due to different flight control policies for UAVs in different regions, the use of the UAV-based monitoring platform must be legal and compliant. On the other hand, the accuracy of the object detection algorithm needs to be improved in future research due to the uniqueness of UAV shooting. Therefore, the future work will adopt recommendations and mainly focus on the following aspects: First, improving the performance of the UAV itself: mainly improving UAV endurance and image transmission. Second, optimizing the flight strategy of UAVs on the road construction site, reducing unnecessary flight and saving electricity. Third, optimizing the target detection algorithm. In the aspect of algorithm optimization, many articles provide ideas concerning ensuring light weight and speed, further improving the accuracy of detection, and automatically recording and saving the detection results.

## Figures and Tables

**Table 1:** UAV basic parameters.

Parameter	Value
Sensor size	12.8 mm × 9.6 mm CMOS
Focal length	10 mm (fixed)
Resolution	5472 × 3648
Weight	907 g
Max. flight time	31 min

**Table 2:** Flight parameters.

Monitoring Area	Altitude (m)	Speed (km/h)	Shooting Area (m × m)
Division	15	50	19.2 × 14.4
Work Zone	10	18	12.8 × 9.6
Cross Area	15	0	19.2 × 14.4

**Table 3:** Training parameters.

Parameters	Value
Pre-trained weight	YOLOv4_weight
Anchor size	Scale 1: [8, 29], [15, 27], [11, 43]
	Scale 2: [16, 51], [18, 68], [23, 64]
	Scale 3: [111, 77], [325, 157], [232, 325]
Freeze training epochs	50
Unfreeze training epochs	250
Batch_size	Freeze: 4
	Unfreeze: 2
Learning rate	10-2

## Data Availability

Not applicable.
